# Prevalence and predictors of ocular complications among children undergoing nephrotic syndrome treatment in a resource-limited setting

**DOI:** 10.1186/s12886-021-01817-6

**Published:** 2021-01-22

**Authors:** Faith Nakubulwa, Rebecca Claire Lusobya, Anthony Batte, Bashir Ssuna, Damalie Nakanjako, Lydia Nakiyingi, Caroline Nalukenge, Francis Onen Sebabi, Ben Mulinde, Juliet Otiti-Sengeri

**Affiliations:** 1 Department of Ophthalmology, School of Medicine, Makerere University College of Health Sciences, Kampala, Uganda; 2 Child Health and Development Centre, Makerere University College of Health Sciences, Kampala, Uganda; 3 Department of Epidemiology and Biostatistics, School of Medicine, Makerere University College of Health Sciences, Kampala, Uganda; 4 Department of Medicine, School of Medicine, Makerere University College of Health Sciences, Kampala, Uganda

**Keywords:** Nephrotic syndrome, Corticosteroids, Ocular complications, Resource limited setting

## Abstract

**Background:**

Nephrotic syndrome is the most common glomerulopathy among children aged 2–18 years and high dose corticosteroids are the backbone of its management. Potentially blinding ocular complications often result from nephrotic syndrome and/or its treatment. We conducted a study to determine the prevalence and predictors of ocular complications among children undergoing nephrotic syndrome treatment at Mulago National Referral Hospital.

**Methods:**

This was a cross-sectional study conducted for three [3] months at the pediatric renal unit of Mulago National Referral Hospital (MNRH). Data from a consecutive sample of 100 children was collected using a semi-structured questionnaire, entered into Epi-data 4.4.2 and exported to STATA 14 for analysis at univariate, bivariate and multivariate levels. A robust Poisson regression model was used to identify predictors of ocular complications.

**Results:**

Out of 100 patients examined, 80(80%) had ocular complications. The median age was 10 (IQR: 7–12) and 52 (52%) were girls. The most frequent complications were hypertrichosis and refractive errors in 71% (95%CI 61.1–79.6) and 56% (95%CI 45.7–65.9) of the patients respectively. Age above 10 years was the predictor for ocular complications with a RR = 1.37 (95%CI:1.14–1.64) *p* = 0.001.

**Conclusions:**

We found a high prevalence of ocular complications among children with nephrotic syndrome in this tertiary hospital. The predictor of ocular complications was age greater than 10 years. We recommend that all children with nephrotic syndrome undergo a baseline ocular examination prior to commencement of treatment and be reviewed periodically by an ophthalmologist.

**Supplementary Information:**

The online version contains supplementary material available at 10.1186/s12886-021-01817-6.

## Background

Nephrotic syndrome (NS) is the most common glomerulopathy in children aged 2-18 years. It puts the patient at a risk of life threatening infections and thromboembolic events if left untreated [1]. Several reports from different settings have indicated variations in the annual incidence of nephrotic syndrome ranging from 2 to 16.7 per 100,000 cases worldwide, with 5.6 per million children in Tropical Africa [2]. In Uganda, the estimated incidence of nephrotic syndrome was at 160 per million population in 2017 [3] . Despite the good therapeutic effects, the use of systemic corticosteroids is associated with multisystem side effects and ophthalmic complications [4]. This has been observed in children who start corticosteroid treatment at a young age, longer duration of therapy [5–7] and a prednisolone dose of 10-15 mg per day for a year has been associated with visually significant cataract [8].

In Mulago National Referral Hospital; Uganda’s national referral hospital, there is currently no baseline ophthalmology screening or follow-up being done for children initiated on corticosteroid treatment for NS and yet over 100 have been managed for the condition according to Mulago Hospital Nephrology department 2018/2019 records. This subjects a large proportion of children to ocular complications, consequently leading to visual disturbances that negatively impact on the quality of life of these children, their performance in school, emotional and social wellbeing. Visual disturbances also translate into a significant socioeconomic burden on family and society due to increased resources needed to raise a visually impaired child [9]. Early detection and prompt management of the side effects can prevent visual deprivation and eventual amblyopia due to cataracts as well as permanent blindness in cases of optic nerve damage in steroid induced glaucoma [10].

It is necessary that patients who are on long term treatment with corticosteroids including those with NS undergo timely examination by an ophthalmologist [1, 11] and prompt referral is instituted for patients at risk of developing steroid-induced glaucoma. Currently, there is a paucity of studies on the occurrence and predictors of ocular complications among children with NS receiving corticosteroids. There are no documented national or hospital guidelines on Ophthalmological screening, management and follow-up of patients with nephrotic syndrome to assess for ocular complications related to the disease and/or its treatment in this resource-limited setting. This study on the prevalence and predictors for ocular complications among children undergoing NS treatment was conducted at a national referral hospital in Uganda, to generate information which may potentially be used by clinicians and policy makers to inform policy and guide development of management and follow-up guidelines for children with NS to reduce ocular complications which subsequently result into visual disturbances. The objectives of this study were to determine the prevalence and predictors of ocular complications among children undergoing nephrotic syndrome treatment at mulago national referral hospital.

## Methods

### Study design and setting

This was a hospital-based, cross-sectional study conducted in Kampala district, Uganda with participants from the pediatric renal unit of Mulago National Referral Hospital (MNRH) between January 2020 and March 2020. The department has both in and outpatient nephrology units which receive patients with nephrotic syndrome all year round who are routinely started on oral corticosteroids as first line of treatment. There is no routine ophthalmology check for these children unless symptomatic.

### Study population

We included all children who had been consented and/ or assented with nephrotic syndrome at MNRH in the pediatric renal unit who were aged 1 to 18 years with documented diagnosis of nephrotic syndrome during the study period. We excluded all children with ocular congenital anomalies and those who were too ill to undergo full ophthalmic exam.

### Data collection procedures and instruments

The study doctor collected data on socio-demographic characteristics, clinical characteristics and treatment characteristics of patients. We used an interviewer-administered questionnaire to collect data on the participants. We then reviewed the file of the participant from the time he or she was admitted up to the current date and recorded the details of the treatment, remissions and relapses. Study-targeted history was collected from the patient followed by physical and ocular examination which was recorded. During ocular examination, visual acuity was measured using an illuminated Snellen chart at 6 m for school going children, Lea test; the gratings for 12-24 months and Cardiff test for 18–60 months. Each eye was occluded in turn and the best visual acuity (VA) assessed. Those found with VA worse than 6/6 had their vision with pinhole assessed and any changes recorded. Visual fields were also tested by the confrontational method. Eye movements were tested in all gazes seeking any paresis or paralysis of extra ocular muscles. Portable slit lamp biomicroscopy was done on all the study participants and findings in the anterior segment recorded. Tonomety was done using a handheld Perkin’s applanation tonometer after the instillation of anesthetic drops (amethocaine) and staining the tear film with fluorescein. The pupils were dilated with cyclopentolate after which a detailed lens examination was done. Wet retinoscopy was done to categorize the refractive errors. Fundus examination was done using an indirect ophthalmoscope and photos taken using Volk iNView Fundus camera and images reviewed by the supervising Ophthalmologist.

Abnormalities found during the ocular examination were documented and children referred to the ophthalmology department for further care.

### Data management and analysis

To determine the prevalence of complications we used a finite sample, at 95% confidence interval and considered a 50% proportion to have complications. While for predictors, we used the Fleiss formula for comparing two proportions and participants were enrolled consecutively.

The collected data was entered into Epidata 4.2 using double entry method, cleaned and exported as a STATA database to STATA 15.1/MP for analysis. The prevalence of ocular complications was determined as a proportion of those who had complications divided by the total number of participants enrolled. The predictors of complications were determined by a robust modified Poisson regression analysis to estimate the risk ratios with their 95% confidence intervals and their *P*-values. At bivariate analysis; clinical and demographic predictors which had a *p* value < 0.2 were included at multivariate level at which only those with a *p* value < 0.05 were considered significant.

## Results

### Participant characteristics

Of the 100 study participants enrolled, 52(52%) were female; the mean age was 10 years (SD 4). Majority of the patients (83%) examined were between 7 and 12 years of age. Majority of the children were in primary school 70(70%) (Table 1).

**Table 1:** Participant characteristics and predictors of ocular complications among children with nephrotic syndrome at Mulago hospital

Characteristic	Total	Proportion(%)	Ocular complications		Crude RR	***P***-value	Adjusted RR(95% CI)	***P***-Value
			No (n, %)	Yes (n, %)				
**Current age in years**								
mean ± SD	10 ± 4							
1–5	17	17	8 (47.1)	9 (52.9)	1		**1**	
6–10	42	42	10 (23.8)	32 (76.2)	1.44	0.138	1.44 (0.89,2.33)	
> 10	41	41	2 (4.9)	39 (95.1)	1.8	**0.012**	**1.37 (1.14,1.64)**	0.001*
**Sex**								
Female	52	52	13 (25.0)	39 (75.0)	1			
Male	48	48	7 (14.6)	41 (85.4)	1.14	**0.195**		
**Residence**								
Rural	34	34	6 (17.7)	28 (82.3)	1			
Semi-urban	32	32	8 (25.0)	24 (75.0)	0.91	0.472		
Urban	34	34	6 (17.7)	28 (82.3)	1.01	0.999		
**Education**								
Pre-school	21	21	9 (42.9)	12 (57.1)	1			
Primary	79	79	11 (13.9)	68 (86.1)	1.51	**0.003**		
**Weight at diagnosis**								
< 20	36	36	10 (27.8)	26 (72.2)	1			
20–29	34	34	7 (20.6)	27 (79.4)	1.1	0.485		
≥ 30	30	30	3 (10.0)	27 (90.0)	1.25	0.068		
**HIV Status**								
Negative	17	17	3 (17.7)	14 (82.3)	1			
Positive	1	1	0	1 (100)	1.21	**0.085**		
Unknown	82	82	17 (20.7)	65 (79.3)	0.96	0.762		
**Current diagnosis**								
In remission	24	24	6 (25.0)	18 (75.0)	1			
NS	62	62	13 (21.0)	49 (79.0)	1.05	0.699		
SDNS	14	14	1 (7.1)	13 (92.9)	1.24	**0.127**		
**Hypertension**								
No	84	84	18 (21.4)	66 (78.6)	1			
Yes	16	16	2 (12.5)	14 (87.5)	1.11	0.332		
**Medication**								
Combination	19	19	3 (15.8)	16 (84.2)	1			
Only prednisolone	81	81	17 (21.0)	64 (79.0)	0.94	0.58		
**Use of traditional eye medicine**								
No	97	97	19 (19.6)	78 (80.4)	1			
Yes	3	3	1 (33.3)	2 (66.7)	0.83	0.65		
**Number of days on treatment**								
< 100	20	20	6 (30.0)	14 (70.0)	1			
100–199	22	22	5 (22.7)	17 (77.3)	1.1	0.598		
≥ 200	58	58	9 (15.5)	49 (84.5)	1.21	0.233		
**Average daily dose of Prednisolone**								
< 15 mg	22	22	3 (13.6)	19 (86.4)	1			
15-30 mg	58	58	14 (24.1)	44 (75.9)	0.88	0.252		
> 30 mg	20	20	3 (15.0)	17 (85.0)	0.98	0.999		
**Cumulative dose per square meter**								
< 5000	61	61	15 (24.6)	46 (75.4)	1			
5000–10,000	27	2	3 (11.1)	24 (88.9)	1.18	**0.101**		
> 10,000	12	12	2 (16.7)	10 (83.3)	1.11	0.503		
**Average daily dose of prednisolone per kg weight**								
< 1	57	57	10 (17.5)	47 (82.5)	1			
1–1.5	30	30	6 (20.0)	24 (80.0)	0.97	0.784		
> 1.5	13	13	4 (30.8)	9 (69.2)	0.84	0.372		
**Number of relapses**								
Infrequent relapse	99	99	20 (20.2)	79 (79.8)	1			
Frequent relapse	1	1	0	1 (100)	1.25	**< 0.001**		

Table 1 shows the characteristics and predictors of ocular complications among children with nephrotic syndrome at mulago national referral hospital

*SD* Standard deviation, *HIV* Human Immunodeficiency Virus, *kg* Kilogram, *NS* Nephrotic syndrome, *SDNS* Steroid dependent nephrotic syndrome

^a^combination = prednisolone + one or more of; cyclophosphamide, mycophenolate mofetil, methotrexate

* *P* < 0.2 at bivariate

*P* < 0.005 at multivariate.

Sixteen children (16%) had history of hypertension. One child out of 18 with confirmed HIV results was HIV positive (Table 1).

### Prevalence of ocular complications

A total of 80/100, 80% (95% CI: 70.8–87.3) participants had ocular complications. Seventy-one (71%) patients had hypertrichosis of the eye lashes, 56 (56%) had refractive errors, 16 (16%) had raised intraocular pressure (IOP) and 1 (1%) had cataract (Table 2).

**Table 2:** Types of ocular complications among children with nephrotic syndrome at Mulago National Referral Hospital

Complications	Frequency (n)	Proportion (%)	95%Confidence Interval
Hypertrichosis	71	71	61.1–79.6
Refractive Errors	56	56	45.7–65.9
Allergic conjunctivitis	37	37	27.6–47.2
Elevated IOP	16	16	9.4–24.7
Bacterial conjunctivitis	10	10	4.9–17.6
Blepharitis	10	10	4.9–17.6
Hordeolums	2	2	0.2–7.0
Corneal scar	1	1	0.02–5.4
Cataract	1	1	0.02–5.4
Optic disc pallor	1	1	0.02–5.4

*IOP* Intraocular pressure

**Fig. 1:**
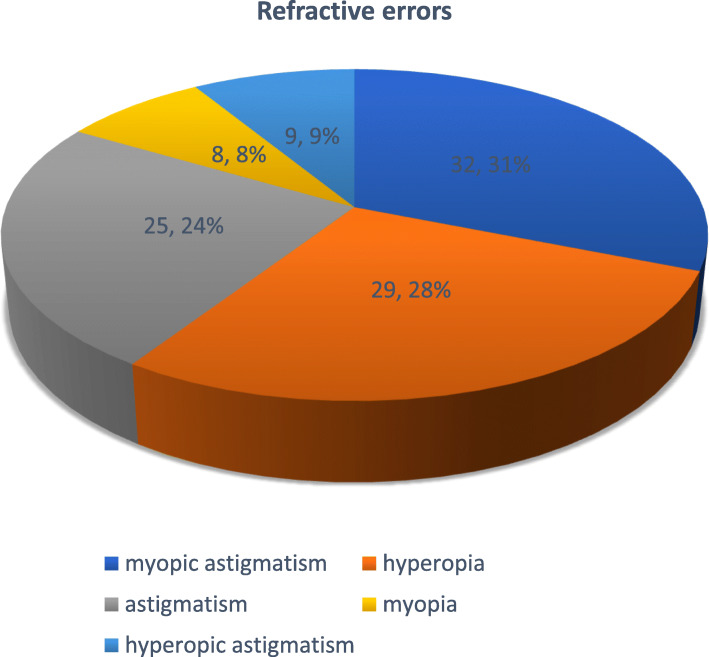
Illustrates the various types of refractive errors among children undergoing nephrotic syndrome treatment; myopic astigmatism was the most common at 32(31%) followed by hyperopia at 29(28%) eyes

Myopic astigmatism was the most common refractive error with 32/103 (31%) eyes. (Fig. 1). The IOP range in the patients with myopic astigmatism was 21-30 mmHg.

### Predictors of ocular complications among children with nephrotic syndrome at Mulago hospital

At bivariate analysis, current age above 10 years (RR = 1.80, *p* = 0.012), a diagnosis of steroid dependent nephrotic syndrome (RR = 1.24, *p* = 0.127), frequent relapses (RR = 1.25, *p* < 0.001), and a cumulative dose per square meter between 5000 and 10,000 mg/m^2^ (RR = 1.18, *p* = 0.101) were statistically significant (*p* < 0.2) and considered for further analysis at multivariate analysis.

At multivariate analysis, current age above 10 years RR = 1.37 (95%CI:1.14–1.64) *p* = 0.001 was the significant predictor of ocular complications among children with nephrotic syndrome.

## Discussion

The prevalence of ocular complications among children with nephrotic syndrome at MNRH was found to be 80%. This was high and probably explained by the high proportion of hypertrichosis among the ocular complications which does not revert in many patients even when medication is stopped [12].

We found no previous studies that stated the prevalence of ocular complications among children with nephrotic syndrome. Majority of the children with NS attending MNRH had one or more ocular complication.

Systemic steroids gain access to the ocular tissues via the blood stream and if taken for a long duration as in NS treatment, their effects on apoptosis, stability of cell membranes, proteolysis and phagocytosis of extracellular matrix contribute to development of the various complications [13–16].

Hypertrichosis was the most frequent ocular complication in this study at 71(71%).Systemic steroids promote facial hypertrichosis through unknown mechanisms [17]. Hypertrichosis has not been reported by previous researchers among children with nephrotic syndrome, however, it was reported among 23 Dutch asthmatic children on inhaled corticosteroids as an adverse effect [12]. Differences occurred in individual susceptibility; occurrence and non-reversal of hypertrichosis and could have been the case for the patients we examined [12].

While Epiblepharon (46.6%) was the most prevalent ocular abnormality among Japanese children with NS, it was not reported among our patients [7]. This was probably because Asians have a more anterior insertion of the levator aponeurosis making epiblepharon prevalent even in their general population [18].

Refractive errors were found in 56 children (56%) and a total of 103 eyes; myopic astigmatism being the most frequent 32(29%). It has been postulated by various researchers that the fluctuating intraocular pressures (IOPs) cause stretching of the globe and transient increases in the axial length resulting in myopic astigmatism, however, no direct correlation has been found [16]. In Kampala, the most prevalent refractive error among primary school children was found to be astigmatism at 52% followed by hyperopia which made our findings different from the general population [19]. Similarly, other studies also found myopic astigmatism as the commonest refractive error among children with nephrotic syndrome. A study by Gheissari et al. found prevalence of myopic astigmatism at 18(24%) [20], Ozaltin recorded myopic astigmatism as the only refractive error among the three children with nephrotic syndrome whom he examined [21] and Agrawal et al. in India found myopic astigmatism as the only refractive error at 5(5%) related to steroid therapy among children with nephrotic syndrome [11].

Elevated IOP was recorded in 16(16%) patients. This was comparable to results from similar studies. Gaur found elevated IOP in 10.97% of the children with Nephrotic syndrome he examined in India [22]. In Japan, 20% of children with nephrotic syndrome had elevated IOP [7]. Agrawal found elevated IOP in 2% of the children with NS that he examined [11]. Corticosteroids which are the mainstay of treatment for nephrotic syndrome cause an elevated IOP by increasing resistance of aqueous outflow at the level of the Trabecular meshwork [14].

This current study found PSC in 1(1%) of the children with NS. This was lower than what other studies found elsewhere. In Japan, 33.3% of the children with NS examined by Hayasaka had PSC while Gaur recorded 26.8% in India, Olonan et al. reported a similarly high occurrence of 13.6% in Manilla Philippines and Wong recorded 10.3% [5, 23]. The difference in the frequency of PSC among the patients with NS in MNRH from those examined elsewhere could not be explained further since this was the first study in an African population. Cotlier proposed that steroids gain entry into the fiber cells of the crystalline lens, react with specific amino acids causing alterations and protein aggregation leading to lens opacification [24].

In our study, children who were 10 years and above had almost twice risk of developing ocular complications than those between 1 and 5 years. This could be because older children had started treatment earlier and therefore had been exposed to the steroids longer than the younger children as reported by Kobayashi et al., 1974 and Brocklebank et al., 1982 [25, 26].

## Conclusions and recommendations

We found a high prevalence of ocular complications among children undergoing nephrotic syndrome treatment in this tertiary hospital. The predictor of ocular complications was age greater than 10 years. We recommend that all children with nephrotic syndrome undergo a baseline ocular examination prior to commencement of treatment and be reviewed periodically by an ophthalmologist.

### Strengths and limitations of the study

It was the first study of this kind in an African population. It is also the only study that has estimated the prevalence of ocular complications among children with nephrotic syndrome. Due to limitation of resources; retinal and choroidal thickening as well as axial length which could have added more meaning to the findings were not measured. Patients were not followed up post steroid therapy to measure residual myopic shift.

## Supplementary Information

**Additional file 1.**

## Data Availability

The datasets used and analyzed during this study are available from the corresponding author on reasonable request.

## Abbreviations

IOP: Intraocular Pressure
MNRH: Mulago National Referral Hospital
NS: Nephrotic syndrome
PSC: Posterior Subcapsular Cataract
SOMREC: School of Medicine Research Ethics Committee
