# Supplementary material for: Prevalence and predictors of ocular complications among children undergoing nephrotic syndrome treatment in a resource-limited setting
Source: BMC Ophthalmol. 2021 Jan 22;21:55. doi: 10.1186/s12886-021-01817-6 (PMC7821477; doi:10.1186/s12886-021-01817-6)
Supplement: Supplementary file 1 — Additional file 1. [file 12886_2021_1817_MOESM1_ESM.docx]

| QUESTIONNAIRE**:** | | | | | | | | | | | | | | | | | | | | | | | | | | | | | | | | | | |
| --- | --- | --- | --- | --- | --- | --- | --- | --- | --- | --- | --- | --- | --- | --- | --- | --- | --- | --- | --- | --- | --- | --- | --- | --- | --- | --- | --- | --- | --- | --- | --- | --- | --- | --- |
| **1) Date**: \|___\|___\| **day** \|___\|___\| **month** \|___\|___\| **yr. Time:___________________** | | | | | | | | | | | | | | | | **2) Patient number: ______________3) Study ID______________** | | | | | | | | | | | | | | | | | | |
| **4) Gender:** □ Male **□**Female | | | | | | | | | | | | | | | | | | | **5) Age:**\|___\|___\| **years**\|___\|___\| **months** | | | | | | | | | | | | | | | |
| **6) Village LCI:** | | | | | | | | | | | | | | | | **7) District:** | | | | | | | | | | | | | | | | | | |
| **8) Religion:** | | | | | | | | | | | | | | | | **9) Tribe:** | | | | | | | | | | | | | | | | | | |
| **10) Relationship with NOK** | | | | |  | | | | | | | | | | | | | | | | | | | | | | | | | | | | | |
| **11) Child’s education** | | | | | □**Not yet** □**Pre-school /Nursery Primary (specify)…………………. Secondary (specify)……………….** | | | | | | | | | | | | | | | | | | | | | | | | | | | | | |
|  | | | | |  | | | | | | | | | | | | | | | | | | | | |  | | | | | | | | |
| **DIAGNOSIS DETAILS** | | | | | | | | | | | | | | | | | | | | | | | | | | | | | | | | | | |
| **12) Initial diagnosis:** □ **Nephrotic syndrome** □**Other** | | | | | | | | | | | | | | | | | | | | | | | | | | **14) Weight at diagnosis ……………………..kg** | | | | | | | | |
| **15) Diagnosis criteria:** □ **Oedema** □ **Urine protein >3+** □ **Low serum albumin** | | | | | | | | | | | | | | | | | | | | | | | | | | | | | | | | | | |
| **16) Date of diagnosis:** \|___\|___\| **day** \|___\|___\| **month** \|___\|___\| **yr.** | | | | | | | | | | | | | | | **17) Time from diagnosis …………………………………………days** | | | | | | | | | | | | | | | | | | | |
| **18) Number of relapses ………………………** | | | | | | | | | | **19) Most recent relapse** \|___\|___\| **day** \|___\|___\| **month** \|___\|___\| **yr.** | | | | | | | | | | | | | | | | | | | | | | | | |
| **20) Diagnosis today:** □**Nephrotic syndrome** □**Steroid dependent nephrotic syndrome** □ **Frequently relapsing nephrotic syndrome**  □ **In remission** | | | | | | | | | | | | | | | | | | | | | | | | | | | | | | | | | | |
| **CURRENT COMPLAINTS** | | | | | | | | | | | | | | | | | | | | | | | | | | | | | | | | | | |
| **SYMPTOM**  **(all must be answered)** | | | **PRESENT** | | | | | | **SYMPTOM**  **(all must be answered)** | | | | | | | | **PRESENT** | | | | | | | | | **SYMPTOM**  **(all must be answered)** | | | | | | | **PRESENT** | |
| **21) Headache** | | | □ Yes | | | □ No | | | **22) Visual hallucinations** | | | | | | | | □ Yes | | | | | □ No | | | | **23) Haloes around bright light** | | | | | | | □ Yes | □ No |
| **24) Eye pain** | | | □ Yes | | | □ No | | | **25) Vomiting** | | | | | | | | □ Yes | | | | | □ No | | | | **26) Reduced vision** | | | | | | | □ Yes | □ No |
| **27) Eyelid swelling** | | | □ Yes | | | □ No | | | **28) Red eyes** | | | | | | | | □ Yes | | | | | □ No | | | | **29) Itching eyes** | | | | | | | □ Yes | □ No |
| **30) Photophobia** | | | □ Yes | | | □ No | | | **31) Tearing** | | | | | | | | □ Yes | | | | | □ No | | | | **32) Spectacle use** | | | | | | | □ Yes | □ No |
| **33) Contact lens use** | | | □ Yes | | | □ No | | | **34) Current eye medication** | | | | | | | | □ Yes | | | | | □ No | | | | **35) Use of traditional eye medication** | | | | | | | □ Yes | □ No |
| **36) Ocular trauma history** | | | □ Yes | | | □ No | | | **37)Family history of open angle glaucoma** | | | | | | | | □ Yes | | | | | □ No | | | | **38) History of connective tissue disorder** □ Yes□ No | | | | | | | | |
| **39) Known to have Diabetes?** | | | | □ Yes | | □ No | | | **40) Known to have hypertension?**□ Yes□ No | | | | | | | | | | | | | | | | |  | | | | | | | | |
| **41) Other history……………………………………………………………………………….** | | | | | | | | | | | | | | | | | | | | | | | | | | | | | | | | | | |
| **MEDICATIONS** | | | | | | | | | | | | | | | | | | | | | | | | | | | | | | | | | | |
| **DRUG** | **Date Started** | | | | | | **Date Stopped** | | | | | **Number of days** | | | | | | | | **Dose (Units)** | | | | | | | | | **Frequency** | | | **Total dose for period** | | |
| **42) Prednisolone** |  | | | | | |  | | | | |  | | | | | | | |  | | | | | | | | |  | | |  | | |
|  |  | | | | | |  | | | | |  | | | | | | | |  | | | | | | | | |  | | |  | | |
|  |  | | | | | |  | | | | |  | | | | | | | |  | | | | | | | | |  | | |  | | |
|  |  | | | | | |  | | | | |  | | | | | | | |  | | | | | | | | |  | | |  | | |
|  |  | | | | | |  | | | | |  | | | | | | | |  | | | | | | | | |  | | |  | | |
|  |  | | | | | |  | | | | |  | | | | | | | |  | | | | | | | | |  | | |  | | |
|  |  | | | | | |  | | | | |  | | | | | | | |  | | | | | | | | |  | | |  | | |
|  |  | | | | | |  | | | | |  | | | | | | | |  | | | | | | | | |  | | |  | | |
|  |  | | | | | |  | | | | |  | | | | | | | |  | | | | | | | | |  | | |  | | |
|  |  | | | | | |  | | | | |  | | | | | | | |  | | | | | | | | |  | | |  | | |
|  |  | | | | | |  | | | | |  | | | | | | | |  | | | | | | | | |  | | |  | | |
|  |  | | | | | |  | | | | |  | | | | | | | |  | | | | | | | | |  | | |  | | |
|  |  | | | | | |  | | | | |  | | | | | | | |  | | | | | | | | |  | | |  | | |
|  |  | | | | | |  | | | | |  | | | | | | | |  | | | | | | | | |  | | |  | | |
|  |  | | | | | |  | | | | |  | | | | | | | |  | | | | | | | | |  | | |  | | |
|  |  | | | | | |  | | | | |  | | | | | | | |  | | | | | | | | |  | | |  | | |
|  |  | | | | | |  | | | | |  | | | | | | | |  | | | | | | | | |  | | |  | | |
| **TOTAL** |  | | | | | |  | | | | |  | | | | | | | |  | | | | | | | | |  | | |  | | |
| **43)Mycophenolate mofetil** |  | | | | | |  | | | | |  | | | | | | | |  | | | | | | | | |  | | |  | | |
|  |  | | | | | |  | | | | |  | | | | | | | |  | | | | | | | | |  | | |  | | |
|  |  | | | | | |  | | | | |  | | | | | | | |  | | | | | | | | |  | | |  | | |
|  |  | | | | | |  | | | | |  | | | | | | | |  | | | | | | | | |  | | |  | | |
|  |  | | | | | |  | | | | |  | | | | | | | |  | | | | | | | | |  | | |  | | |
| **44)Tacrolimus** |  | | | | | |  | | | | |  | | | | | | | |  | | | | | | | | |  | | |  | | |
|  |  | | | | | |  | | | | |  | | | | | | | |  | | | | | | | | |  | | |  | | |
|  |  | | | | | |  | | | | |  | | | | | | | |  | | | | | | | | |  | | |  | | |
| **45)Furosemide** |  | | | | | |  | | | | |  | | | | | | | |  | | | | | | | | |  | | |  | | |
|  |  | | | | | |  | | | | |  | | | | | | | |  | | | | | | | | |  | | |  | | |
|  |  | | | | | |  | | | | |  | | | | | | | |  | | | | | | | | |  | | |  | | |
| **46)Levamisole** |  | | | | | |  | | | | |  | | | | | | | |  | | | | | | | | |  | | |  | | |
|  |  | | | | | |  | | | | |  | | | | | | | |  | | | | | | | | |  | | |  | | |
|  |  | | | | | |  | | | | |  | | | | | | | |  | | | | | | | | |  | | |  | | |
| **47)Enalapril** |  | | | | | |  | | | | |  | | | | | | | |  | | | | | | | | |  | | |  | | |
|  |  | | | | | |  | | | | |  | | | | | | | |  | | | | | | | | |  | | |  | | |
|  |  | | | | | |  | | | | |  | | | | | | | |  | | | | | | | | |  | | |  | | |
| **48)Captopril** |  | | | | | |  | | | | |  | | | | | | | |  | | | | | | | | |  | | |  | | |
| **49)Amlodipine** |  | | | | | |  | | | | |  | | | | | | | |  | | | | | | | | |  | | |  | | |
| **50)Carvedilol** |  | | | | | |  | | | | |  | | | | | | | |  | | | | | | | | |  | | |  | | |
| **51)Bisoprolol** |  | | | | | |  | | | | |  | | | | | | | |  | | | | | | | | |  | | |  | | |
| **Other medicines** |  | | | | | |  | | | | |  | | | | | | | |  | | | | | | | | |  | | |  | | |
| **52)** |  | | | | | |  | | | | |  | | | | | | | |  | | | | | | | | |  | | |  | | |
| **53)** |  | | | | | |  | | | | |  | | | | | | | |  | | | | | | | | |  | | |  | | |
| **54)** |  | | | | | |  | | | | |  | | | | | | | |  | | | | | | | | |  | | |  | | |
| **55)** |  | | | | | |  | | | | |  | | | | | | | |  | | | | | | | | |  | | |  | | |
|  | | | | | | | | | | | | | | | | | | | | | | | | | | | | | | | | | | |
| **MOST RECENT INVESTIGATIONS** | | | | | | | | | | | | | | | | | | | | | | | | | | | | | | | | | | |
| **Renal function tests** | | | | | | | | **Complete blood count** | | | | | | | | | | | | | | | | | | | **Urinalysis** | | | | | | | |
| **56) Creatinine ………………… (mg/dl)**  **57) Na+ ……………………………. (mmol/l)**  **58) K+ ……………………………. (mmol/l)**  **59) Cl^-^……………………………. (mmol/l)**  **60) Ca^2+^ ……………………………. (mmol/l)**  **61) TCO_2_ ……………………………. (mmol/l)**  **62) Glu ………………… (mg/dl)**  **63) BUN ………………… (mg/dl)**  **64) HCT ……………………………(%)**  **65) Anionic gap……………………(mmol/l)** | | | | | | | | **66) WBC Count …………………………………. X10^3^/µl**  **67) Neutrophil count……………………………. X10^3^/µl**  **68) Lymphocyte count ……………………………. X10^3^/µl**  **69) RBC count…………………………………. X10^3^/µl**  **70) Haemoglobin ……………………………g/dl**  **71) Haematocrit ……………………..%**  **72) MCV …………………………………….fl**  **73) PLT…………………………………. X10^3^/µl**  **OTHER TESTS**  **74) HIV: □Positive □Negative**  **75) ANA: □Positive □Negative** | | | | | | | | | | | | | | | | | | | **76) Proteins………………….**  **77) RBCs ………………………**  **78) SG ……………………………**  **79) PH …………………………….**  **80) Nitrites ………………………**  **81) Ketones ………………………..**  **82) Bilirubin …………………………**  **83) Urobilinogen ……………………….**  **84) Glucose ………………………………**  **LIVER FUNCTION TESTS.**  **Albumin……………………….g/dL** | | | | | | | |
| **BLOOD TESTS TODAY** | | | | | | | | | | | | | | | | | | | | | | | | | | | | | | | | | | |
| **85) Random blood sugar …………………………..mmol/l 86) Time since last meal ……………………………………………** | | | | | | | | | | | | | | | | | | | | | | | | | | | | | | | | | | |
| **87) Glycosylated haemoglobin: …………………………………%** | | | | | | | | | | | | | | | | | | | | | | | | | | | | | | | | | | |
| **EXAMINATION** | | | | | | | | | | | | | | | | | | | | | | | | | | | | | | | | | | |
| **GENERAL EXAMINATION** | | | | | | | | | | | | | | | | | | | | | | | | | | | | | | | | | | |
| **88) Weight ……………………………….Kg** | | | | | | **89) Height ………………………m** | | | | | | | | | | | | | | | | | **90) Pulse ……………………………./min** | | | | | | | | | | | |
| **91) Blood pressure** | | | | | | **1. …………/………………mmHg (Arm □Lt □Rt) 2. …………/………………mmHg (Arm □Lt □Rt)**  **3. …………/………………mmHg (Arm □Lt □Rt) Average ………………/……………mmHg**  **Percentile……….** | | | | | | | | | | | | | | | | | | | | | | | | | | | | |
| **OCCULAR EXAMINATION** | | | | | | | | | | | | | | | | | | | | | | | | | | | | | | | | | | |
| **92) Visual Acuity: RE…………………… LE……………………… WITH PINHOLE: RE…………………… LE………………………** | | | | | | | | | | | | | | | | | | | | | | | | | | | | | | | | | | |
| **93) Intraocular pressure: RE…………………………….(mmHg) LE……………………………………..(mmHg)** | | | | | | | | | | | | | | | | | | | | | | | | | | | | | | | | | | |
| **94) Retinoscopy findings: □ Myopia □ Hyperopia □ Astigmatism □ Emmetropia**  **□ Other: Specify …………………………………..** | | | | | | | | | | | | | | | | | | | | | | | | | | | | | | | | | | |
| **Eyelids** | | **95) Blepharitis** □ Yes□ No  **96) Chalazion** □ Yes□ No  **97) Entropion** □ Yes□ No | | | | | | | | | | | | | | | | | | | **98) Epiblepharon**□ Yes□ No  **99) Stye** □ Yes□ No  **100) Ectropion** □ Yes□ No | | | | | | | | | | | | | |
| **Eyelashes** | | **101) Trichiasis:** □ Yes□ No  **102) Hypertrichosis:** □ Yes□ No | | | | | | | | | | | | | | | | **103) Dystichiasis** □ Yes□ No | | | | | | | | | | | | | **104) Absent:** □ Yes□ No | | | |
| **Conjunctiva** | | **105) Hyperaemic:** □ Yes□ No | | | | | | | | | | | | **106) Growth:** □ Yes□ No | | | | | | | | | | | | | **107) Allergic conjunctivitis:** □ Yes□ No | | | | | | | |
| **Cornea** | | **108) Clear:** □ Yes□ No | | | | | | | | | | | | | | | | | | | **109) Corneal Ulcer:** □ Yes□ No | | | | | | | | | | | | | |
| **Anterior chamber** | | **110) Formed:** □ Yes□ No | | | | | | | | | | | **111) Deep:** □ Yes□ No | | | | | | | | | | | | | | | | | | **112) Quiet:** □ Yes□ No | | | |
| **Pupil** | | **113) Round:** □ Yes□ No | | | | | | | | | | | **114) Reactive:** □ Yes□ No | | | | | | | | | | | | | | | | | **115) Regular:** □ Yes□ No | | | | |
| **Lens** | | **116) Natural lens:** □ Yes□ No | | | | | | | | | | | **117) Cataract:** □ Yes□ No | | | | | | | | | | | **118) If yes which type………………………………..** | | | | | | | | | | |
| **Vitreous** | | **119) Clear:** □ Yes□ No **comment**………………………………. | | | | | | | | | | | | | | | | | | | | | | | | | | | | | | | | |
| **Fundus** | | **120) Optic nerve head**  □ **Normal □ Abnormal**  **Specify abnormality………………………** | | | | | | | | | | | | | **121) Normal blood vessels**  □ Yes□ No comment:……………….. | | | | | | | | | | | | | **122) Features of hypertension/Diabetic retinopathy**  □ Yes□ No | | | | | | |
| **Eye movement** | | **123)** H H **(Tick appropriately)** | | | | | | | | | | | | | **Comment on defect ………………………………………………………………** | | | | | | | | | | | | | | | | | | | |
| **Visual fields** | | 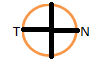**124) RE:** | | | | | | | | | 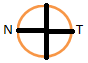**LE:** | | | | | | | | | | | | | | **Comments ………………………………………………………………………………………………………………………………………………………………………………………………………………..** | | | | | | | | | |

‘
